# Molecular Fingerprinting of the Omicron Variant Genome of SARS-CoV-2 by SERS Spectroscopy

**DOI:** 10.3390/nano12132134

**Published:** 2022-06-21

**Authors:** Antonio Alessio Leonardi, Emanuele Luigi Sciuto, Maria Josè Lo Faro, Dario Morganti, Angelina Midiri, Corrado Spinella, Sabrina Conoci, Alessia Irrera, Barbara Fazio

**Affiliations:** 1 Dipartimento di Fisica e Astronomia “Ettore Majorana”, Università degli Studi di Catania, Via S. Sofia 64, 95123 Catania, Italy; antonio.leonardi@dfa.unict.it (A.A.L.); mariajose.lofaro@dfa.unict.it (M.J.L.F.); 2 CNR-IMM Catania University, Istituto per la Microelettronica e Microsistemi, Via S. Sofia 64, 95123 Catania, Italy; 3 Lab SENS CNR, Beyond NANO, Viale Ferdinando Stagno d’Alcontres 31, 98166 Messina, Italy; emanueleluigi.sciuto@unime.it (E.L.S.); corrado.spinella@cnr.it (C.S.); sabrina.conoci@unime.it (S.C.); 4 Dipartimento di Scienze Chimiche, Biologiche, Farmaceutiche, ed Ambientali, Università degli Studi di Messina, Viale Ferdinando Stagno d’Alcontres 31, 98166 Messina, Italy; dario.morg@hotmail.it; 5 Dipartimento di Patologia Umana, Università di Messina, Via Consolare Valeria 1, (Azienda Ospedaliera Universitaria Policlinico “G. Martino”), 98125 Messina, Italy; angelina.mediri@unime.it; 6 CNR-IMM Istituto per la Microelettronica e Microsistemi, Zona Industriale, VIII Strada 5, 95121 Catania, Italy; 7 CNR-IPCF, Istituto per i Processi Chimico-Fisici, Viale F. Stagno D’Alcontres 37, 98158 Messina, Italy

**Keywords:** SERS, SARS-CoV-2, omicron variant, Ag dendrites

## Abstract

The continuing accumulation of mutations in the RNA genome of the SARS-CoV-2 virus generates an endless succession of highly contagious variants that cause concern around the world due to their antibody resistance and the failure of current diagnostic techniques to detect them in a timely manner. Raman spectroscopy represents a promising alternative to variants detection and recognition techniques, thanks to its ability to provide a characteristic spectral fingerprint of the biological samples examined under all circumstances. In this work we exploit the surface-enhanced Raman scattering (SERS) properties of a silver dendrite layer to explore, for the first time to our knowledge, the distinctive features of the Omicron variant genome. We obtain a complex spectral signal of the Omicron variant genome where the fingerprints of nucleobases in nucleosides are clearly unveiled and assigned in detail. Furthermore, the fractal SERS layer offers the presence of confined spatial regions in which the analyte remains trapped under hydration conditions. This opens up the prospects for a prompt spectral identification of the genome in its physiological habitat and for a study on its activity and variability.

## 1. Introduction

The severe acute respiratory syndrome coronavirus 2 (SARS-CoV-2) has continued to spread worldwide since December 2019, sowing pandemic infections that have affected over 500 million people and caused more than 6 million global deaths so far. The high transmissibility of the virus impacted our lives in many ways, with serious consequences on health, social relationships, and the global economy. Furthermore, its continuous mutations, a typical process to which all viruses are subjected, have generated the rapid succession of numerous variants that have increased the ease of contagion and sometimes escaped the scrutiny of diagnostic tests and the responses of the human immune system, thus making the vaccines less effective [1]. For these reasons, a rapid detection and, at the same time, a thorough understanding of the characteristics of the circulating variant is desirable in order to quickly undertake the social measures for public health.

Generally, early diagnosis of viral infections unveils as crucial for both limiting pandemic development and providing effective treatments. The current tests based on reverse transcription–polymerase chain reaction (RT-PCR) [2,3,4,5] are highly reliable, but require long times for processing, expensive equipment, and expert personnel. Moreover, these assays are able to detect virus variants, but do not distinguish them, consequently requiring their sequencing with expensive and even very time-consuming methods as well [6,7,8]. On the other hand, the lateral flow tests are commonly used as a rapid assay to tackle the pandemic spread but offer a lower reliability than PCR [9]. Indeed, in the case of several SARS-CoV-2 variants of concern, such as the Omicron, these lateral flow tests present impaired detection with a reduced reliability. Nowadays, new optical methods are emerging in modern biosensing as alternatives to these rapid detection platforms [10,11,12,13], as they are able to increase both sensitivity and specificity, aiming to meet the recommendations of the World Health Organization, requiring at least 97% of specificity and a minimum sensitivity of 70% for rapid tests.

In particular, the optical methods based on the inelastically scattered light in spontaneous Raman processes [14] represent a very powerful answer for nondestructive and standoff label-free unique identification and structural characterization of materials. Raman spectroscopy boasts applications ranging from material science [15,16,17,18] to the detection of hazardous materials [19,20], from biological science up to medical diagnosis and clinical implementations [21,22,23,24]. In principle, Raman spectroscopy is capable of revealing SARS-CoV-2, whatever the variant. Moreover, it is also known to provide the characteristic spectral fingerprint depending on the variant type [25], overcoming the limit of standard diagnostic tests. Additionally, this vibrational spectroscopy, when applied to single-stranded(ss) RNA and DNA studies, enables to distinguish genomic alterations, as pioneered by the works of J.G. Kelly et al., who discriminated genomic variability such as the methylation pattering [26,27]. This approach can provide great interest in the case of SARS-CoV-2, as the virus seems to epigenetically silence some genes [28].

However, due to the intrinsically low cross-section, spontaneous Raman scattering only provides very weak signals. This can represent a disadvantage, especially in the biomedical field, where there is the need to reveal very small traces of biological species present in bodily fluids, whose detection is a key point of help to enable scientists to provide the early diagnosis of important diseases.

Recently, a great scientific effort has been put into conceiving different methods to enhance the Raman signal in order to take full advantage of its potential, such as the surface-enhanced Raman scattering (SERS) [29,30]. SERS, indeed, is becoming an excellent tool for sensing of biological species. Different methods have been implemented for SERS biosensing, with strategies including performing plasmonic supports [31,32,33] in the liquid environment [34,35], implementing selectivity [36], and combining immunoassay approaches [37,38]. In particular, SERS approaches to SARS-CoV-2 have mainly dealt with the selective detection of the spike protein. Chen H. and coworkers obtained a sensitive detection of the virus down to 10 PFU/mL by means of its recognition of the DNA aptamer receptor incubated on Au nanopopcorn surfaces [39]. An assay for widespread use in the rapid identification of the SARS-CoV-2 infections by means of a single chain variable fragment (scFv) of recombinant antibody has been also demonstrated [40]. In this case, the scFvs were conjugated to SERS nanotags and bound to the spike protein, for which a limit of detection (LOD) of 1 or 2 orders of magnitude lower than the viral load in an infected person was obtained. However, all of these experiments did not take advantage of implementing Raman spectroscopy for the molecular fingerprint identification of the virus, as only a change to the SERS spectrum of the receptor is detectable.

In the present study, we exploited the potential of SERS to unveil, for the first time to our knowledge, the vibrational fingerprints of the genome of the SARS-CoV-2 Omicron variant. At present, taking into account the sequences reported to GISAID [41], Omicron is the dominant variant of concern in circulation because it shows increased transmission and reinfection with respect to the Beta and Delta variants [42]. For the above purpose, we used a three-dimensional (3D) plasmon platform based on silver dendrites, known to allow for the detection of biomolecules in hydration conditions [33]. The SERS enhancement allows us to obtain and identify the Raman spectrum of the single-stranded ribonucleic acid (ssRNA) of the SARS-CoV-2 Omicron species in very short acquisition times. This enables for a rapid and univocal discrimination of this variant through vibrational evaluations that can enrich the new vibrational library of the “Raman genome”, as defined by Pezzotti et al. [25]. Moreover, this methodology can pave the way towards a rapid detection of the epigenetic variability of the virus.

## 2. Materials and Methods

### 2.1. SARS-CoV-2 Genome Preparation

The RNA genome of the SARS-CoV-2 Omicron variant was provided by the Department of Human Pathology of the University Hospital of Messina (Italy). The genome, i.e., a single-stranded RNA molecule of 29,793 bases, was extracted from a swab sample, resuspended in RNase-free H_2_O, and directly used in the Raman analysis.

In parallel, the RNA was quantified by RT-PCR, by which the RNA was converted into complementary DNA copies (cDNA) and amplified in real time, reporting a cycle threshold (Ct) value of about 19.5. This value was, then, used to calculate the exact number of genome copies per µL (cps/µL), according to the mathematic conversion reported by Brandolini et al. [14], which gave a final concentration of 10^4^ cps/µL (corresponding to ~0.2 nM, as reported in the Section S1 of Supplementary Materials). A drop of 100 µL of the RNA genome solution (10^6^ effective copies) was then spotted on top of an Ag dendrites sample, incubated at 50 °C for 4 h, and left dry in air at room temperature before the Raman measurements. The analysis was also performed directly on the genome solution.

### 2.2. Ag Dendrites: Chemicals, and Structural and Optical Measurements

Fractal silver dendrites were produced by wet chemical etching of a commercial silicon wafer (Siegert Wafer, Charlottenburger Allee 7, 52068 Aachen, Germanygert wafer Allee 7, 52068 Aachen, Germany), 4 inches diameter and 500 μm thick, and n-type doped (1–5 Ω·cm). The chemical solution was a combination of silver nitrate (AgNO_3_) 0.05 N by Scharlau (Scharlau Turkey, Solen Residence A Blok No: 19/4 Ic Kapi No: 109 Tasocagi Yolu Cad. Mahmutbey, Istanbul, Turkey) and hydrofluoric acid (HF) 40% by Sigma-Aldrich (2:1 *v*/*v*) in deionized water.

Ag dendrite fractal morphology was imaged by means of a ZEISS Supra 25 Scanning Electron Microscope (Carl-Zeiss-Straße 22 73447 Oberkochen, Germany) in cross section configuration. The extinction measurements were performed by a UV–VIS Perkin-Elmer spectrometer (Perkin Elmer, Waltham 940 Winter St, Waltham, MA, USA) equipped with an integrating sphere and used in diffuse reflectance configuration, because the transmission configuration was forbidden due to the silicon substrate strongly absorbing in the visible range. The reflectance spectrum was then converted in apparent absorbance (extinction) obtained as log(100/R%).

### 2.3. Raman and SERS Measurements

The Raman spectrum of 10^4^ cps/µL of the genome of the SARS-CoV-2 Omicron variant in liquid water and then the SERS spectrum of the same genome solution drop casted on the Ag dendrite platform were acquired by means of a micro-Raman spectrometer (LabRAM HR800) from Horiba/Jobin-Yvon (Kyoto, Japan) equipped with an 1800 lines/mm grating and a Peltier cooled CCD detector (Synapse model from Horiba). The radiation from an argon-ion laser (Spectra-Physics 2060, Spectra-Physics LASER OPTRONIC S.r.l.-North Via Quaranta, 57 20139 Milano, Italy) at the wavelength of 514.5 nm was focused onto the samples by a 100X (0.9 NA) objective mounted on an Olympus microscope BX41(Olympus Italia S.r.l. Via San Bovio 1-3, 20054, Segrate, Italy), and then collected in the backscattering configuration with integration times of 30 s for all the spectra here reported. The Raman spectrum of the SARS-CoV-2 Omicron genome was performed by focusing a laser power of 2.5 mW into a glass microcell containing 75 μL of the solution, consisting of microscope slides with a hemispherical cavity (15–18 mm diameter and 0.5–0.8 mm depth), purchased from Marienfled GmbH, and covered with a microscope glass coverslip (Forlab).

For the SERS measurements, we focused a low laser power (25 μW) on the sample in order to minimize the plasmonic heating effects [43] and avoid the damage of the investigated biomaterial. Since the SERS spectra notoriously variate [44], we used the following protocol. We acquired each spectrum for 30 s to average the blinking of the spectral signals of the molecules, performing a kind of “random walk” on the metal surface [45], especially in the absence of a functionalization. Indeed, the blinking mechanism usually ranges in a timescale of seconds [46]. Moreover, to increase the reliability of our measurements, we acquired spectra in six different points of the central region of the sample, where we spotted the solution drop. The spectra always reveal the same fingerprints of the SARS-CoV-2 RNA genome with slightly different variations between them, confirming the reproducibility of our experiments. SERS spectra have been acquired in a wide Raman frequency range (200–4000 cm^−1^); Voigt and Gaussian profiles have been adopted to separate the vibrational contributions and fit the experimental data between 2500 and 4000 cm^−1^.

## 3. Results and Discussion

### 3.1. Synthesis and Characterization of Ag Dendrites

Three-dimensional (3D) materials are establishing themselves as the most efficient plasmonic substrates, as they exploit a very high surface-to-volume ratio and increase active SERS sites. The higher density of “hot spot” regions along the third direction (vertical) with respect to a flat substrate (2D) guarantees an increased sensitivity. Among the fabrication techniques of 3D materials, metal-assisted chemical etching (MACE) stands out as a low-cost and industrially compatible process, whose purpose is generally the realization of silicon nanowires [47,48,49,50,51,52,53,54,55,56,57]. This approach, indeed, allows for a dense forest of silicon nanowires (Si NWs) whose length can be tuned and whose large surface can be decorated with other materials [47,58], such as metal nanostructures. This occurrence permits realizing very sensitive 3D SERS substrates [59,60,61]. Furthermore, during the fabrication process of Si NWs by means of MACE, silver dendrites are obtained as a waste product of the chemical reaction, representing good candidates for highly sensitive SERS performances [33,59].

**Figure 1:**
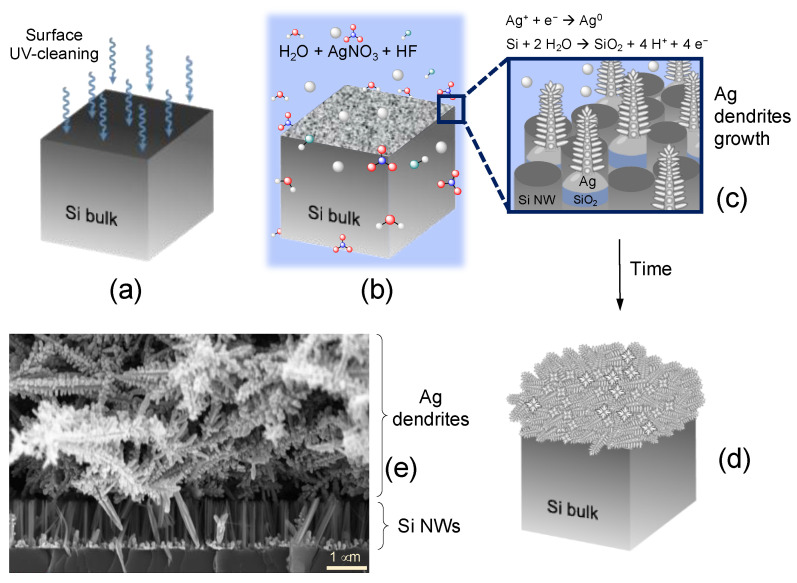
Scheme for the realization of silver dendrite fractal system by metal-assisted chemical etching. (**a**) The Si wafer is first treated for the removal of organic contamination by UV–ozone cleaning, for the removal of native oxide,, and then (**b**) etched in an AgNO_3_/HF solution. During the etching, (**c**) the formation of Ag dendrites occurs as a byproduct of the reaction, (**d**) and the final effect is the formation of a dense film of silver fractal dendrites. (**e**) Cross-section scanning electron microscopy of the fractal silver dendrites lying on top of Si nanowire structure.

The schematic of the Ag dendrite synthesis flow that we adopted in this work is shown in Figure 1.

We first cleaned the silicon surface by UV–ozone treatment for 2 min in order to remove organic contamination (Figure 1a). Subsequently, we removed the native oxide by etching the silicon wafer in a 5% HF aqueous solution. Finally, we dipped the silicon substrate in a deionized water solution containing hydrofluoric acid and silver nitrate (see Section 2 for details), as depicted in Figure 1b. The silver salt dissolves in aqueous solution, releasing the Ag^+^ ions, which are reduced to metallic silver (Ag^0^) at the silicon–substrate interface. The reaction driving force lies in the difference between the electrochemical potentials of the redox pairs Ag^+^/Ag^0^ and Si^0^/SiO_2_. The reduction potential of silver is more positive than the Fermi energy of the silicon substrate [62], so the electrons from silicon to Ag^+^ are transferred. The reduction of Ag^+^ to metallic silver at the Si interface occurs by forming nuclei, as pictured in Figure 1c [63] (cathodic reaction), and, simultaneously, the silicon oxidizes in H_2_O (anodic reaction). With increasing time, the Ag nuclei grow into silver nanoparticles randomly distributed on the silicon surface. The just-formed silicon oxide under the Ag nanoparticles is chemically etched by the HF present in the solution, and the nanoparticles sink into the Si substrate. These nanoparticles seed the nucleation of the incoming additional silver, allowing for the formation of Ag dendrites at the equilibrium condition onto the Si surface (Figure 1c,d) and without a capping agent [64,65].

The size and morphology of the synthesized Ag structures were examined by scanning electron microscopy (SEM) and reported in Figure 1e.

**Figure 2:**
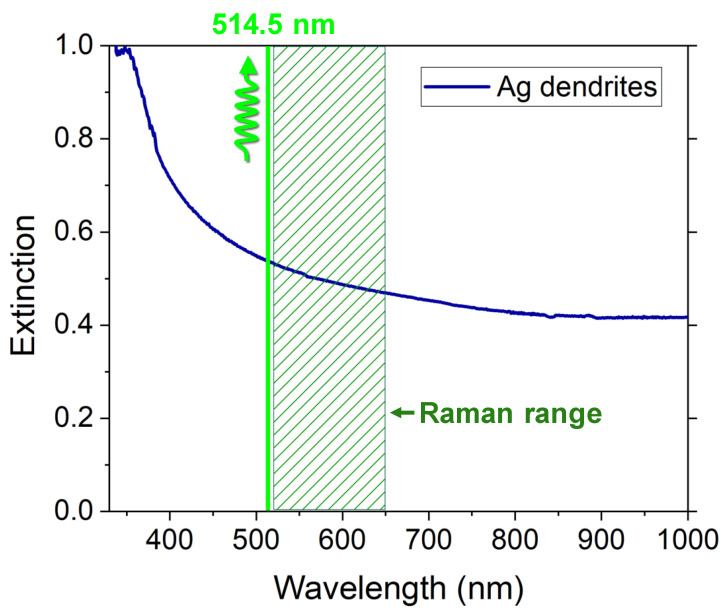
Extinction of the Ag dendrites in the visible NIR range. The laser wavelength used for SERS excitation at 514.5 nm and the corresponding Raman region of SARS-CoV-2 Omicron variant analyzed in this work (520–648 nm, corresponding to 200–4000 cm^−1^), are indicated by the vertical green line and the dark green dashed box, respectively.

In Figure 2, we show the extinction spectrum of the as-prepared 3D Ag dendritic material. We observe a wide and efficient plasmon band extending through the visible range up to the near infrared region. This large resonance is due to the material fractal shape, characterized by self-similarity and scale invariance properties that lead to inhomogeneities of all sizes, and, as a consequence, to fluctuations of the refractive index on the whole length scale [66,67]. When light interacts with these inhomogeneities, whatever the wavelength, it will be multiply scattered resonant with them, and the plasmon resonance will be always matched. This is a great advantage in SERS measurements, for which not only the exciting wavelength (green line in Figure 2) but also the extended range of Raman frequency modes (box green sparse-filled in Figure 2) fall within the plasmon resonance band to take advantage of the huge enhancement.

Moreover, random fractal patterns open up the opportunity to achieve a complex multiscale disorder where unexpected and fascinating physical phenomena frequently emerge, related to multiple scattering and interference. This generates a strong scattering that can lead to electromagnetic field localizations, resulting in the formation of hot-spot regions [68,69,70].

Additionally, the presence of nanocavities on the whole length scale, where a drop-casted watery solution can remain trapped in confined regions, allows for the spectroscopic investigations of biomolecules of interest in hydration conditions with respect to their physiological habitat [33].

### 3.2. Identification of Raman Vibrational Fingerprints of Genome of SARS-CoV-2 Omicron Variant

**Figure 3:**
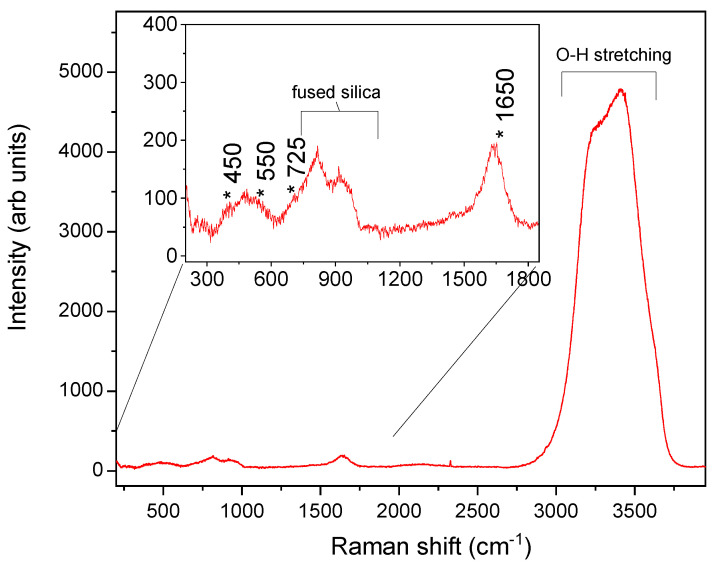
Raman spectrum of the Omicron variant of SARS-CoV-2 in RNase-free H_2_O, where we can distinguish the vibrational contributions of water only. In the inset, the 300–1800 cm^−1^ range is highlighted.

The Raman spectrum of 10^4^ cps/µL (0.2 nM) of the genome of the Omicron variant of SARS-CoV-2 in the RNase free water is shown in Figure 3, where we clearly recognize all the contributions of the O-H vibrations of the water molecules only, whereas no vibrational signals coming from SARS-CoV-2 are detectable. In particular we distinguish the O-H stretching modes in the region between 3000 and 3800 cm^−1^, the H-O-H bending vibration at 1650 cm^−1^, and the variety of intermolecular optic modes as the three librations between 330 and 1000 cm^−1^. As shown in the highlighted spectral region reported in the inset of Figure 3, we recognize (1) the libration around the two-fold H_2_O axis at about 450 cm^−1^, (2) the in-plane (rocking) libration at 550 cm^−1^, and (3) the out-of-plane libration at 725 cm^−1^, the latter of which overlapped to the contribution between 800 and 1000 cm^−1^ by the fused silica of the coverslip for the glass microcell containing the liquid solution.

**Figure 4:**
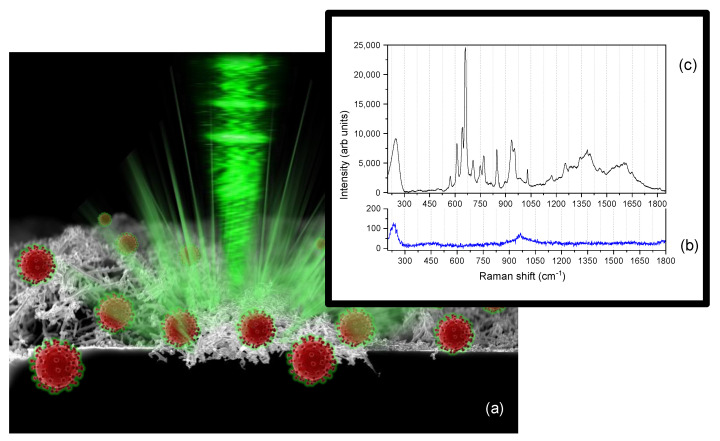
Sketch of the SERS experiment: a green laser is focused onto the 3D silver dendrite layer, where a drop of solution containing 0.21 nM of SARS-CoV-2 Omicron genome in RNase-free water was spotted (**a**). A strong plasmonic effect is activated due to the presence of strong electromagnetic enhancement at the nanogaps, therefore making visible the Raman spectrum of the Omicron variant in its natural habitat. (**b**) SERS spectrum of bare Ag dendrites. (**c**) SERS spectrum of the Omicron variant of the SARS-CoV-2.

In order to identify the Raman vibrational fingerprints of the Omicron SARS-CoV-2 ssRNA, we then had to resort to SERS experiments by using Ag dendrites to improve the spectral signal of the analyte (see Section 2 for details). A schematic depiction of the SERS platform is given in Figure 4a. It should be noted that the inability to detect the spontaneous Raman signal prevents us from correctly assessing the SERS enhancement factor (EF) of the silver dendrites when the excitation wavelength is set at 514.5 nm, as in this experiment. However, since in previous work we have attested that the EF of this platform is greater than six orders of magnitude at the excitation wavelength of 633 nm [33], we assume that the EF can also show higher values in the current measurements based on the shape of the plasmon resonance band previously shown in Figure 2.

We first acquired the SERS spectrum of the bare Ag dendrite (Figure 4b), which appears characterized by the strong peak of the Ag-O species at 235 cm^−1^ [71], and by the peak of the S-O vibrational mode at 962 cm^−1^ due to the reaction with oxygen during the synthesis process and sulfur contamination when exposed to air, respectively [72]. The other Ag-O vibrational band at 430 cm^−1^ [71], typical of silver oxidation during the UV cleaning procedure in an ozone rich environment [33], is just visible in the spectrum, and organic contamination is not present in the spectral region between 1000 to 1600 cm^−1^. When adding pure water (RNase-free) to the plasmonic substrate, we only detect the O-H stretching vibrations typical of the network of H_2_O molecules in the range 3000–3800 cm^−1^, as shown in Figure S1 (Section S2) of Supplementary Materials [33]. This occurrence attests to the confinement of liquid water molecules in the nanocavities of the dendrites. On the other hand, it guarantees to detect any no other contaminating species present in the plasmonic material or in the water itself. When the genome watery solution (~0.2 nM) is drop-casted into the Ag dendrite SERS platform (Figure 4c), the peak at 235 cm^−1^ shifts to higher Raman frequencies up to 245 cm^−1^. This is typical of the formation of an Ag-N chemical bond between the silver and nitrogen groups of the adsorbed biomolecules, which vibrates at frequencies very close to the Ag-O bond [73], thus causing a broadening of the SERS band and a shift at its maximum intensity. Despite the complexity of the spectral signal due to the contributions of multiple conformers present in the ssRNA that generally make it difficult to decipher, in Figure 4c we can distinguish the typical fingerprints of RNA nucleobases, purines (adenine (A) and uracil (U)), and pyrimidines (guanine (G) and cytosine (C)), and its backbone.

**Figure 5:**
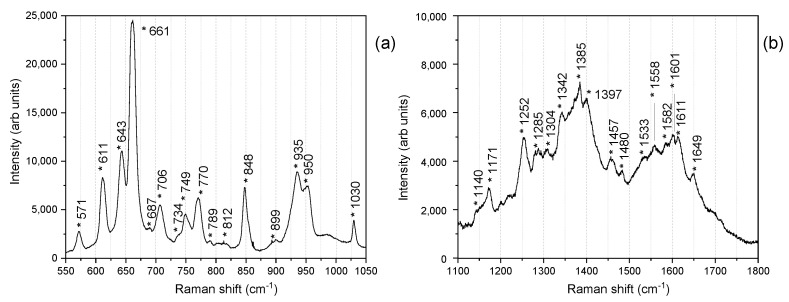
Details of the acquired genome SERS spectrum: (**a**) the spectral region between 550 and 1050 cm^−1^ and (**b**) range between 1100 and 1800 cm^−1^.

Figure 5a shows a zoom in the spectral region between 550 and 1050 cm^−1^, which is dominated, in the lower frequency zone, by the intense peaks mainly related to the symmetric stretching modes and the deformations of nucleobases and ribose rings in the nucleosides. In particular, the most intense band at 661 cm^−1^ is assigned to the ring breathing mode of guanine, while the analogous modes related to adenine at 734 cm^−1^, and cytosine and uracil overlapping at 789 cm^−1^ [74,75], show less intense Raman peaks. The dominating intensity of the guanine peak may be due to the preferential conformations observed for the guanosine [76], which may play a key role when laying in the proximity of a SERS metal surface. In the SERS effect, indeed, the electrical field is strongly enhanced when polarized along the nanocavities axes [77,78], where hot-spot regions form, thus allowing the molecules with polarizability tensors along the axes directions to experience the highest enhancement effect [79]. Being in the Ag dendrites, the nanocavities are randomly oriented, and we can imagine the guanosine preferentially disposing flat along these axes.

Moreover, the G band at 661 cm^−1^ is a marker of the A-form secondary structure of the ssRNAs, having only residual B-type backbone conformations, whose marker is the G band at 684 cm^−1^ [80].

Other vibrational peaks due to in-plane ring deformations of nucleic acids are assigned (571, 610, 643, 687, 704, and 770 cm^−1^). The spectral range between 820 and 1050 cm^−1^ is characterized by vibrations of the sugar–phosphate backbone, as witnessed by the bands located at 848 and 900 cm^−1^ [76,81], and by the sharp peak of the bending mode of the ribose rings at 1030 cm^−1^ [74,82]. We also distinguish the peaks of the ring deformations of the nucleobases at 935 and 954 cm^−1^ [75].

The range between 1100 and 1800 cm^−1^ (Figure 5b) shows overlapped peaks due to the in-plane ring vibrations (C-N stretching) of the nucleic acids and carbonyl stretching modes [74,75,80].

**Figure 6:**
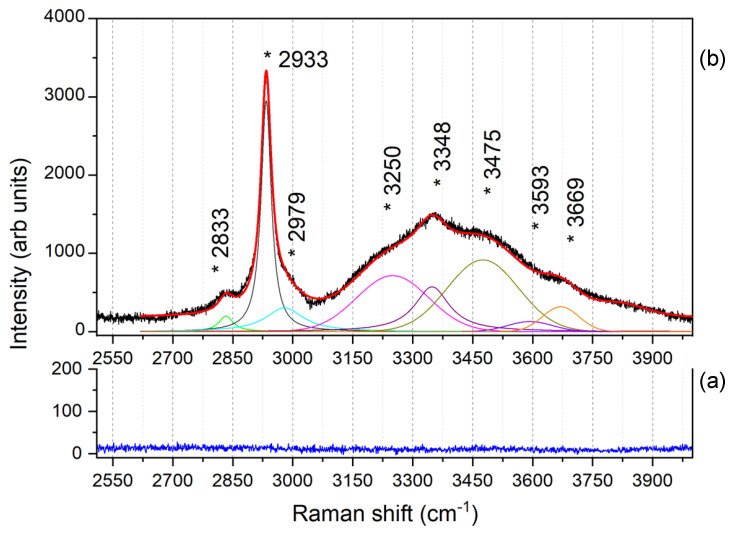
Comparison between the (**a**) reference spectrum of the featureless bare Ag dendrites (blue line) and (**b**) that one of ssRNAs of the SARS-CoV-2 Omicron variant (black line) in the region between 2500 and 4000 cm^−1^, fitted (red line) considering the contributes described in the text. The colored lines in (**b**) are the fitting curves separating the different spectral contributions. In particular, the green-, dark grey-, and cyan-colored lines represent the C-H stretching contributions; the magenta (at 3250 cm^−1^), the dark yellow (at 3475 cm^−1^), and the violet (at 3593 cm^−1^) lines represent the network, intermediate, and multimers water bands, respectively, as described in the text. The purple and orange lines indicate the N-H stretching band of nucleobases (at 3348 cm^−1^) and the ribose O-H stretching band (at 3669 cm^−1^), respectively.

The high-frequency Raman range in the region between 2500 and 4000 cm^−1^, where the fingerprint of the O-H stretching vibrations is usually detected, is shown in Figure 6. Here, we compare the reference spectrum of the bare Ag dendrites (a), which appears devoid of any spectroscopic feature, with that one of the ssRNAs of the SARS-CoV-2 Omicron variant (b), for which a fitting procedure have been adopted to separate the vibrational contributions.

The C-H stretching modes vibrating between 2700 and 3100 cm^−1^ (green, dark grey, and cyan lines) are typical of the RNA spectrum and come mainly from the C-H groups of the ribose [83]. Between 3000 and 4000 cm^−1^, we find the complex spectrum formed by the O-H vibration bands of both the RNA (nucleobases and sugars) and the interfacial water molecules [84]. In particular, we notice the rearrangement in the hydrogen bond network typical of liquid water that give rise to three bands with relative intensities typical of interfacial water. In particular, we find the so-called band of “network water” at 3250 cm^−1^ (magenta line), representing the highest degree of connectivity of the H-bond, that of the “intermediate water” at 3475 cm^−1^ (dark yellow line), representing a distorted network, and that of the “multimers water” (dimers or trimers) around 3593 cm^−1^ (violet line) [33,85]. Furthermore, the N-H stretching bands of nucleobases are distinguishable at 3348 cm^−1^ (purple line) [86], and the ribose O-H stretching band at 3669 cm^−1^ (orange line) [87], whereas the other O-H vibrations, due to the sugar in the RNA backbone, are not identifiable, as they are mixed with those of the interfacial water.

The tentative assignment of the vibrational peaks for all the extended Raman frequency range is reported in Table 1.

We emphasize that our findings show that, during the SERS experiments, the SARS-CoV-2 molecules are hydrated, laying in a liquid environment. Indeed, the morphology of the Ag dendrite SERS substrate allows for the presence of nanocavities, where very small volumes of liquid solution remain enclosed. Moreover, these confined regions are subjected to very strong plasmonic effects due to the generation of hot spots. It is noteworthy that the acquisition of the SERS spectra of the biomolecules from the hot spots in a liquid environment is a desired goal, due to its enormous potential and the opportunity to access molecular activity without incurring conformational changes and the denaturation occurring when analytes are dried on the surface of the SERS substrates. In this latter case, in fact, whatever the enhancement ability of the engineered hot spots, SERS, although capable of providing the ultrasensitive detection of a target, may not be able to gain information on the molecular activity and interaction with the environment.

## 4. Conclusions and Perspectives

In this work, we provide the first Raman vibrational fingerprint of the genome of the SARS-CoV-2 Omicron variant, with the aim of expanding the Raman library of virus variants [25]. To achieve this goal, we have resorted to the SERS properties of a particular type of plasmonic material based on silver dendrites, which allow not only a high amplification of both the incident and the Raman scattered electromagnetic fields on a large scale of frequencies, but also provide access to the molecular activity in its physiological environment. Further studies will be devoted to exploiting the notable sensing ability of this SERS platform. Therefore, we plan both to suitably functionalize the Ag platform surface to selectively capture SARS-CoV-2 and to explore the limit of detection in this circumstance. We conclude by commenting that the Raman spectroscopy offers a rapid recognition of the type of variant of concern and potentially enables to distinguish the epigenetic alterations of the virus. These occurrences, combined with the detection capability of the SERS technique, would therefore be strategic so to understand how to promptly block the infection activity of the virus and to undertake social measures for public health.

## Figures and Tables

**Table 1:** Detailed identification of each Raman mode in comparison with the literature and assignment.

Raman Frequency Shift (cm^−1^)		Mode Assignment *
SERS-This Work	[References]	
571	574 [75] 577 [76]	Guanine ring in-plane deformation In-plane υ_sym_ Ribose ring in Guanosine
611	617 [76]	Guanine out-of-plane def. ω (C-N-C) in guanosine
643	646 [76]	Uracil and Ribose ring in-plane ρ in uridine
661	660 [75] 665 [74]	Guanine ring breathing in Guanosine
687	689 [75]	Out-of-plane Adenine ring deformation
706	704 [75]	Cytosine ring deformation
734	734 [75] 733 [74] 735 [76]	Adenine ring breathing in Adenosine
749	753 [74]	Not assigned vibrations in Cytidine
770	776 [76] 764 [75]	Out-of-plane Uracil ring deformation
789	786 [74]	Cytosine and Uracil ring breathing in nucleosides
812	814 [88]	O-P-O υ_sym_ in RNA backbone
848	848 [81]	Ribose stretching
899	902–906 [76]	In-plane Ribose ring stretching (C-C) in nucleosides
935	924 [75]	In-plane Adenine ring deformation
950	957 [75]	In-plane Guanine ring deformation
1030	1032 [74] 1043–1048 [76]	Ribose ring bending in nucleosides
1140	1133–1143 [76]	In-plane υ_sym_ (N-C-N, C=C) in nucleosides
1171	1183–1190 [76]	Ribose ring deformation in nucleosides
1252	1256 [76] 1251 [75]	Adenine υ (C-N)
1285	1275 [75]	Guanine υ (C-N)
1304	1307 [75] 1302 [76]	Cytosine υ (C-N)
1342	1341 [76] 1335–1347 [76]	Adenine υ (C-C, C=N) in adenosine Guanine υ (N-C=C, N-C=N) in guanosine
1385	1381 [76] 1382 [75]	Out-of-plane ρ/υ in Ribose ring (C-C, HOCH_2_) Guanine ring stretching C-N
1397	1401 [76]	Uracil υ_asym_ (C-N-C, C-C) and Ribose ring in-plane υ in Uridine
1457	1436–1467 [76]	Cytidine and Adenine in-plane υ and Ribose ring ρ in nucleosides
1480	1482 [75]	Cytosine υ (C-N)
1533	1532 [75]	Uracil in-plane υ (C-C, C-N)
1558	1553 [75]	Adenine in-plane δ (NH_2_)
1582	1584 [76]	Guanine in-plane υ_asym_ (N-C=C, N-C=N); υ (C=O); ρ (C-N) in guanosine
1601	1608 [76]	Adenine in-plane υ_asym_ (N-C=C); in-plane υ (C=C); δ (H-N-H) in Adenosine
1611	1612 [76]	Cytosine in-plane υ (C=C, C=O); in-plane υ_asym_ (C=C-N); δ (H-N-H) in Citidine
1649	1649 [76]	Guanine in-plane υ (C=O); in-plane υ_asym_ (N=C-C); δ (H-N-H) in Guanosine
2833	2800–3050 [83]	Ribose υ (C-H) groups in RNA
2933		
2979		
3250	3295 [85]	υ (O-H) in bulk water
3348	3356 [86]	υ (N-H) in pyrimidine bases
3475	3460 [85]	υ (O-H) in distorted network
3593	3590 [85]	υ (O-H) in multimer water
3669	3654 [87]	υ (O-H) in Ribose

* υ, stretching; υ_sym_, symmetric stretching; υ_asym_, asymmetric stretching; ω, wagging; ρ, rocking; δ, scissoring.

## Data Availability

Data is contained within the article or Supplementary Material.

## Supplementary Materials

The following supporting information can be downloaded at: https://www.mdpi.com/article/10.3390/nano12132134/s1.
