# Supplementary material for: Molecular Fingerprinting of the Omicron Variant Genome of SARS-CoV-2 by SERS Spectroscopy
Source: Nanomaterials (Basel). 2022 Jun 21;12(13):2134. doi: 10.3390/nano12132134 (PMC9268696; doi:10.3390/nano12132134)
Supplement: Supplementary file 1 [file nanomaterials-12-02134-s001.zip › nanomaterials-1753155-supplementary.pdf]

Supplementary Material

Molecular fingerprinting of the Omicron variant genome of SARS-CoV-2 by SERS spectroscopy

Antonio Alessio Leonardi [1,2], Emanuele Luigi Sciuto [3,4], Maria Josè Lo Faro [1,2], Dario Morganti[4], Angelina Midiri [5], Corrado Spinella [3,6], Sabrina Conoci [3,4,6], Alessia Irrera [3,7]* and Barbara Fazio [3,7]*

1 Dipartimento di Fisica e Astronomia "Ettore Majorana", Università degli studi di Catania, Via S. Sofia 64, 95123, Catania, Italy.
2 CNR-IMM Catania University, Istituto per la Microelettronica e Microsistemi, Via S. Sofia 64, 95123, Catania, Italy.
3 Lab SENS CNR, Beyond NANO, Viale Ferdinando Stagno d'Alcontres 31, 98166, Messina, Italy.
4 Dipartimento di Scienze Chimiche, Biologiche, Farmaceutiche, ed Ambientali, Università degli studi di Messina, Viale Ferdinando Stagno d'Alcontres 31, Messina, 98166 Italy.
5 Dipartimento di Patologia Umana, Università di Messina, Via Consolare Valeria 1, (Azienda Ospedaliera Universitaria Policlinico "G. Martino") 98125, Messina, Italy.
6 CNR-IMM Istituto per la Microelettronica e Microsistemi, Zona Industriale, VIII strada 5, Catania, 95121 Italy
7 CNR-IPCF, Istituto per i Processi Chimico-Fisici, Viale F. Stagno D'Alcontres 37, 98158, Messina, Italy.
* Correspondence: A.I. irreraalessia@gmail.com; B.F. barbara.fazio@cnr.it

Citation: Leonardi, A.A.; Sciuto, E.L.; Lo Faro, M.J.; Morganti, D.; Midiri, A.; Spinella, C.; Conoci, S.; Irrera, A.; Fazio, B. Molecular Fingerprinting of the Omicron Variant Genome of SARS-CoV-2 by SERS Spectroscopy. *Nanomaterials* **2022**, *12*, 2134. https://doi.org/10.3390/nano12132134

Academic Editor: Vincenzo Amendola

Received: 17 May 2022
Accepted: 18 June 2022
Published: 21 June 2022

Publisher's Note: MDPI stays neutral with regard to jurisdictional claims in published maps and institutional affiliations.

Copyright: © 2022 by the authors. Licensee MDPI, Basel, Switzerland. This article is an open access article distributed under the terms and conditions of the Creative Commons Attribution (CC BY) license (https://creativecommons.org/licenses/by/4.0/).

Section S1. Calculation of the SARS-CoV-2 genome concentration in molarity

The final concentration in cps/µL was converted in molarity, whose calculation is made based on the assumption that the average molecular weight of a single nucleotide is 325 Daltons.

- Eq.: N. copies = (DNA concentration (ng/µl) x [6.022 x 10^{23}]) / (length of template x [1x10^{9}] x 325).
- DNA concentration = N. copies x length of template x [1x10^{9}] x 325 / (6.022 x 10^{23}) = 0.002 ng/µL.

This concentration was adopted to calculate the molarity by using the molecular weight of the entire SARS-CoV-2 Omicron genome (9536 g/mol) and obtaining a value of ~ 0.2 nM.

Section S2. SERS spectrum of water molecules confined in Ag dendrites substrate

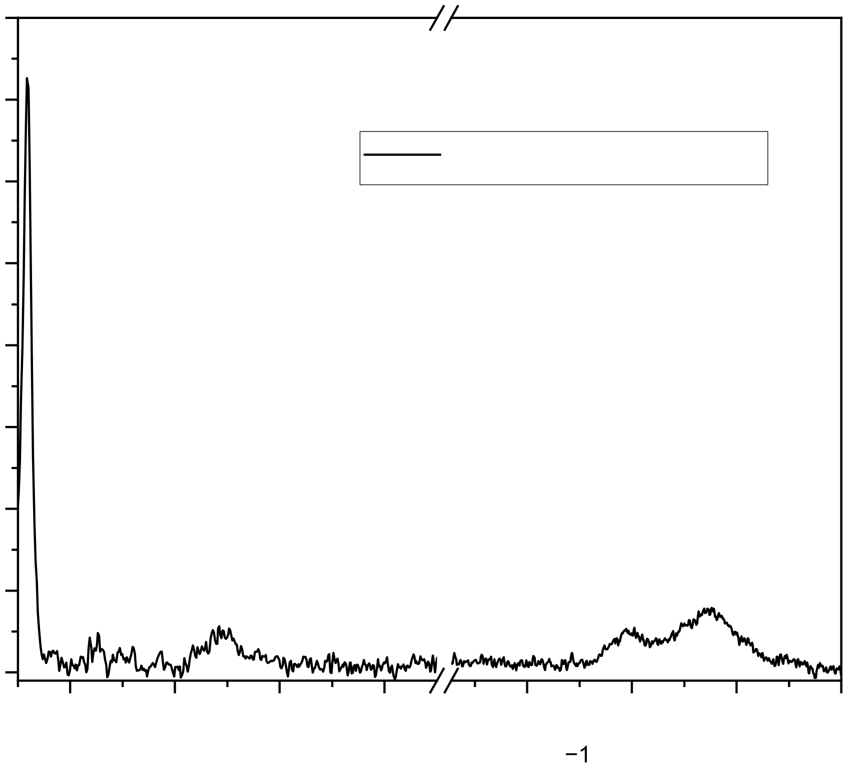

Figure S1. SERS spectrum of water molecules confined in Ag dendrites nanocavities. We distinguish the strong peak of Ag-O species at 235 cm^{-1} and the peak of the S-O vibrational mode at 962 cm^{-1} due to the sulfur contamination of the substrate when exposure to air. The only spectral contributions assigned to the sample added to the plasmonic material are the OH stretching vibrations, in the region between 3000 and 3800 cm^{-1}, due to the water molecules organized in a liquid network. The water molecules, indeed, remain confined in the liquid state inside the nanocavities of dendrites, as commented in the main text. Moreover, the lack of any other spectral signature highlights the absence of contaminations coming from the plasmonic substrate or from the water that we used to prepare the biological solutions.
